# Electrodermal synchrony of patient and therapist as a predictor of alliance and outcome in psychotherapy

**DOI:** 10.3389/fpsyg.2025.1545719

**Published:** 2025-05-14

**Authors:** Wolfgang Tschacher, Eugénia Ribeiro, Alexandra Gonçalves, Adriana Sampaio, Pedro Moreira, Joana Coutinho

**Affiliations:** ^1^University Hospital of Psychiatry and Psychotherapy, University of Bern, Bern, Switzerland; ^2^Psychology Research Center (CIPsi), School of Psychology, University of Minho, Braga, Portugal

**Keywords:** physiological synchrony, electrodermal activity, therapeutic alliance, cognitive-behavioral therapy, patient-leading, session outcome

## Abstract

Previous empirical psychotherapy studies have been analyzing patient-therapist physiological synchrony as an important marker of an adaptive interpersonal co-regulation process between patient and therapist, and therefore a process essential for developing the therapeutic alliance. Yet, research on synchrony of the electrodermal activity and its relationship with the therapeutic alliance is still scarce, with inconsistent findings. The present study aimed to analyze the association between electrodermal synchrony, a signature of coordinated sympathetic activation, and the quality of the therapeutic alliance throughout the therapeutic process. Twenty-one therapeutic dyads were recruited, consisting of 21 different patients with a diagnosis of major depressive disorder or social anxiety disorder, treated by six therapists. For each dyad, electrodermal activity was recorded during all 16 sessions of Cognitive-Behavioral Therapy. The Working Alliance Inventory and the Session Evaluation Questionnaire were administered at each session to monitor the quality of the alliance and the respective session. Symptomatic improvements were measured with outcome questionnaires. We found clear evidence for the presence of in-phase electrodermal synchrony of therapists and patients. Additional results were that patient-leading synchrony was significantly more pronounced than therapist-leading synchrony, and that this leading role of patients in their sympathetic interactions during sessions was positively linked with the quality of the therapeutic bond as rated by the therapist, and negatively linked with patients’ distress.

## 1 Introduction

Previous studies have shown that in psychotherapy not only the verbal exchange between patient and therapist is effective, but also their bodily behavior and its synchronization, i.e., the reciprocal temporal entrainment of the therapist’s and patient’s activity. In line with these results, a growing body of research systematically addresses the role of synchrony in psychotherapy, postulating its existence in several biobehavioral modalities (Koole and Tschacher, 2016; Zilcha-Mano, 2024). Evidence for the presence of synchrony was found for patients’ and therapists’ body movements as well as various physiological parameters (Palumbo et al., 2017; Tschacher and Meier, 2020).

Various studies showed associations between such nonverbal synchrony and self-reported therapeutic alliance and outcome in different therapeutic approaches (Altmann et al., 2020) and different manifestations for depression and anxiety disorders (Paulick et al., 2018). Whereas in several studies interpersonal synchrony was found associated with good therapeutic results (e.g., Paz et al., 2021; Ramseyer and Tschacher, 2011), findings have remained somewhat inconsistent overall (Paulick et al., 2018; Jennissen et al., 2024; Gregorini et al., 2025). By now, there is ample evidence that synchrony commonly emerges in the therapeutic dyad, in other dyads such as couples (Coutinho et al., 2019) and even in interacting strangers (Tschacher et al., 2014; Coeugnet et al., 2025), but it is not always associated with favorable results. For example, vocal pitch synchrony may be related to ruptures in the therapeutic alliance (Reich et al., 2014).

Hence, synchrony may reflect adaptive processes of emotional co-regulation, but may also reflect intensive oppositional (“anti-phasic”) interactions such as anger escalation processes and increased autonomic reactivity to the other’s negative affect as tend to occur in conflictual and competitive interactions. Therefore, in order to understand whether synchrony indicates a co-regulation process or a co-dysregulation process, in which both partners feel threatened and incapable of regulating themselves, we must analyze not only the level of synchrony (high versus insignificant), but also its polarity (positive covariation of the two partners’ physiological responses in the same direction—in-phase—versus negative covariation in opposite directions—anti-phase). In addition, synchrony between two individuals need not be symmetrical, as one participant in the dyad may obtain a leading role in the co-regulation process (Kupper et al., 2015).

Nonverbal synchrony of body movement has been the type of synchrony most often addressed in the psychotherapeutic context owing to the unobtrusiveness of motion capture from video recordings. Physiological synchrony assessing the coordination of physiological time series of the two interacting partners has come to the fore increasingly in recent years, when physiological monitoring became more economical and less invasive. Physiological markers in psychotherapeutic contexts have captured researchers’ attention also because of their value for identifying subtle changes in affect and arousal. Physiology holds the promise of representing the biological basis of emotional and cognitive processes that may be activated without the therapist’s and patient’s awareness (Deits-Lebehn et al., 2020). Tschacher and Meier (2020) found positive associations between physiological synchrony, particularly of cardiac activity indicators, and assessments of the therapeutic alliance and session outcome. Empirical work has emerged focusing on physiological synchrony between patient and therapist (Avdi and Evans, 2020) and its potential association with psychotherapeutic common factors (e.g., Kleinbub et al., 2020) such as the therapeutic alliance quality. Researchers have analyzed the relationship between these variables in individual therapy contexts (Bar-Kalifa et al., 2019) as well as in couple therapy contexts (Tourunen et al., 2020; Kykyri et al., 2025). Overall, this literature suggested that greater synchrony of electrodermal activity was linked with positive qualities of the relationship such as empathy (Marci et al., 2007; Messina et al., 2013). According to Koole and Tschacher (2016), synchrony is associated with the therapeutic alliance, contributing to its establishment, given that synchrony allows access to internal states of the other in the course of mutual alignment.

Electrodermal activity (EDA) is a physiological signal of specific relevance as it provides a direct signature exclusively of the sympathetic, arousal-related branch of the autonomous nervous system. EDA thus differs from the other peripheral physiological signals in that it is not a mixture of sympathetic and parasympathetic/vagal components. EDA is measured by the changes in electrical conductivity of the skin surface, which directly corresponds to the amount of sweat secreted by the eccrine sweat glands located in the hypodermis of the surfaces of the hand. Given that the activity of the eccrine glands is uniquely controlled by the sympathetic nervous system, conductivity provides a valid and reliable measure of sympathetic activation alone. Therefore, the increase in conductivity and hence EDA reflects an increase in sympathetic activation (Del Piccolo and Finset, 2018; Palumbo et al., 2017), which is recognized as one of the most sensitive markers of arousal associated with emotional and cognitive processing, both conscious and non-conscious. EDA increase indicates emotional activation, including the experience of emotions such as anger, fear, or joy, whereas its decrease is associated with states of passivity, such as ease and collapse. Other than the more complex cardiac or respiratory measures, EDA can be used as a linear and strong index of emotional activation, signaling the saliency of emotional events that take place within the psychotherapeutic interaction.

Marci et al. (2007) analyzed the association between EDA synchrony, empathy and socio-emotional processes during the therapeutic process. Their findings suggested that higher levels of EDA synchrony reflected a greater sharing of cognitive and emotional states of arousal within the therapeutic dyad. Additionally, they showed that the dyad exhibited more responses of positive consideration in a context of high EDA synchrony compared to moments of low synchrony. According to Ham and Tronick (2009), when an individual actively meets the needs of another, the probability of synchronization of electrodermal activity is higher. Correspondingly, the increase in emotional distance within the therapeutic dyad (e.g., change in gaze and attention) can lead to a decrease in the synchrony of electrodermal activity (Marci and Orr, 2006). In a more recent work by Gernert et al. (2023), higher levels of EDA synchrony were associated with a decrease in the severity of symptomatology presented by the patient. Likewise, Bar-Kalifa et al. (2019) explored the association between the synchrony of electrodermal activity in the therapeutic dyad and the therapeutic alliance, finding that synchrony was positively associated with the “bond” component of the therapeutic alliance. However, this result was only present during segments of an emotion-focused technique, but not during segments of more traditional cognitive-behavioral techniques.

An investigation in the context of couple therapy found that EDA synchrony had some associations with the therapeutic alliance, yet the two phenomena did not completely overlap (Tourunen et al., 2020). The results of this study suggested that individuals in a group context can be impacted by the relationships established between other individuals. For example, one of the associations found by Tourunen and colleagues was that, faced with an increase in physiological synchrony between the male patient and the therapist (also male), the assessments of the therapeutic alliance carried out by the spouse (i.e., the female patient) likewise showed an increase. In the context of couple therapy, Coutinho et al. (2023) found evidence for the presence of EDA synchrony for the patient-therapist and therapist-therapist dyads but not patient–patient dyads, highlighting the challenges involved in synchrony computation in multi-person settings. This study suggested that patients’ well-being as well as therapists’ alliance ratings were significant predictors of patient–patient EDA synchrony.

Despite the growing research interest in this topic, the empirical results concerning the presence of synchrony and its relationship with outcome and/or process measures are still inconsistent. In our view, this inconsistency may be due to non-linear relationships between synchrony and measures of dyadic functioning such as the alliance within the context of psychotherapy. It may be true that excessive levels of physiological synchrony during the psychotherapeutic session may derive either from relational conflict or the therapist not promoting adaptive interactions (Tschacher et al., 2023; Riess, 2011). Another explanation for inconsistent results may be the still small number of studies with sufficient statistical power and the heterogeneity of methods used for the acquisition and processing of physiological measures as well as for the final computation of synchrony (Altmann et al., 2022). More specifically, most previous research did not consider the *polarity* of synchrony, in other words did not distinguish between in-phase (positively correlated) and anti-phase (negatively correlated) synchronized behavior. Such ‘traditional’ synchrony research was based, as a default, on the *absolute* values of cross-correlations, thus treating cross-correlations with negative sign as positive. This however rules out detection of anti-phase synchrony, which means that, in the course of social interaction, high values in one person significantly coincide with low values in the other and vice versa, indicating sort of a turn-taking behavior. Awareness of this shortcoming was raised since empirical findings indicated that anti-phase synchrony is found, e.g., in heart-rate synchrony (Tschacher and Meier, 2020) and eye movement synchrony (Tschacher et al., 2021).

Furthermore, research to date has largely neglected to study the potential *asymmetry* of dyads, although this may provide important information especially when the dyad members have different roles such as therapists and patients in psychotherapy. One might expect that therapists have a leading role because, especially in the theory of behavioral psychotherapies, therapists are considered ‘experts’ and should take the lead. Moreover, who assumes the leading role in interactions was found associated with the patients’ psychopathology (Kupper et al., 2015), which makes this aspect of synchrony additionally relevant for psychotherapy.

Thus, and in the light of inconsistencies within synchrony research, the present study aimed to provide additional empirical data to clarify the role of physiological synchrony for therapeutic alliance and outcome, using a sample with sufficient statistical power and a valid biological measure. In addition, we wished to go into the details of the synchrony phenomenon by including information on the polarity of synchrony and the potential leader-follower asymmetry. We selected EDA as the measure of interest due to its unique relationship with sympathetic arousal.

The goals of the present study were four-fold, consisting of one hypothesis and three research questions of an exploratory nature. The fundamental analysis concerned the evidence for the presence of EDA synchrony throughout the sessions of cognitive-behavioral psychotherapy. Our hypothesis 1 was that, in line with previous research, synchrony would be significantly found in therapy sessions. The second question was exploratory: Is there predominant in-phase or anti-phase synchrony? In addition to this information on the polarity of covariation, we also wished to analyze the direction of influence within the dyad, that is, whether it was the patient who tended to lead the synchrony process, with the therapist following, or the other way around. Third, we wished to explore whether there was a significant association between EDA synchrony and alliance and further outcome measures in our dataset. Fourth, in all these analyses we considered the two different variants of synchrony computation, the ‘traditional’ synchrony algorithm based on absolute cross-correlations and the synchrony algorithm that distinguishes between in-phase and anti-phase polarities.

## 2 Methods

### 2.1 Participants

The sample consisted of 21 patients and six therapists, selected from the dataset of the ColPsi research project. To be included in the study, participants had to be over the age of 18, and be diagnosed with either an anxiety disorder or major depressive disorder. All participants had provided written informed consent prior to inclusion. Individuals who met any of the following criteria were excluded from the study: Comorbidity with other Axis I disorders, current substance abuse, presence of suicidal ideation or severe mental disorder. Of the 21 patients, 14 (66.7%) were female and seven male, with ages ranging from 18 to 52 years (M = 31.52, SD = 11.21). Fifteen participants (71.4%) had been diagnosed with major depressive disorder and six (28.6%) with social anxiety disorder. Therapists (five females and one male) adopted a cognitive-behavioural approach, and had professional experience varying between 4 and 22 years (M = 10.33, SD = 6.26).

### 2.2 Measures

#### 2.2.1 Self-report measures

##### 2.2.1.1 Working alliance inventory (WAI)

The WAI contains three subscales according to the conceptualization of Bordin (1979): agreement on tasks, agreement on goals, and development of a therapeutic bond, each subscale consisting of 4 items rated on 5-point Likert-type scales (1 = “rarely” to 5 = “always”). Higher scores thus indicate a stronger therapeutic alliance. In the present study both the therapist and the patient versions of the WAI (short revised version) were used. The WAI shows good validity and reliability (Horvath et al., 1989; Hatcher and Gillaspy, 2006).

##### 2.2.1.2 Session outcome

The Session evaluation questionnaire (SEQ) assesses the quality of each session in four dimensions: depth, smoothness, positivity and arousal. The first two dimensions evaluate the session process, whereas the last two evaluate the participants’ post-session mood and activation. The SEQ includes 21 items, rated using a 7-point bipolar adjective format. Higher scores indicate higher depth, smoothness, positivity, or arousal (Stiles, 1980). Additionally, the brief outcome questionnaire OQ-10.2, a condensed version of the OQ-45.2, was employed at sessions 2 to 15, with subscales wellness and distress.

##### 2.2.1.3 Therapy outcome

The Outcome Questionnaire OQ-45.2 (Machado and Fassnacht, 2015) was applied once at the beginning and at the termination of the 21 therapy courses, at sessions 1 and 16. Since the clinical group displayed two distinct affective disorders, major depression or social anxiety disorder, a transdiagnostic measure was used for the assessment of psychological distress. The OQ-45.2 provides an assessment of symptomatic change across the course of the psychotherapeutic treatment, with three subscales: social role, distress and interpersonal relations. In two therapies that were terminated early the post measurement was omitted, so that only 19 global outcome measures based on the OQ-45.2 were available.

#### 2.2.2 Physiological measures

##### 2.2.2.1 Acquisition of electrodermal activity (EDA)

**Figure 1:**
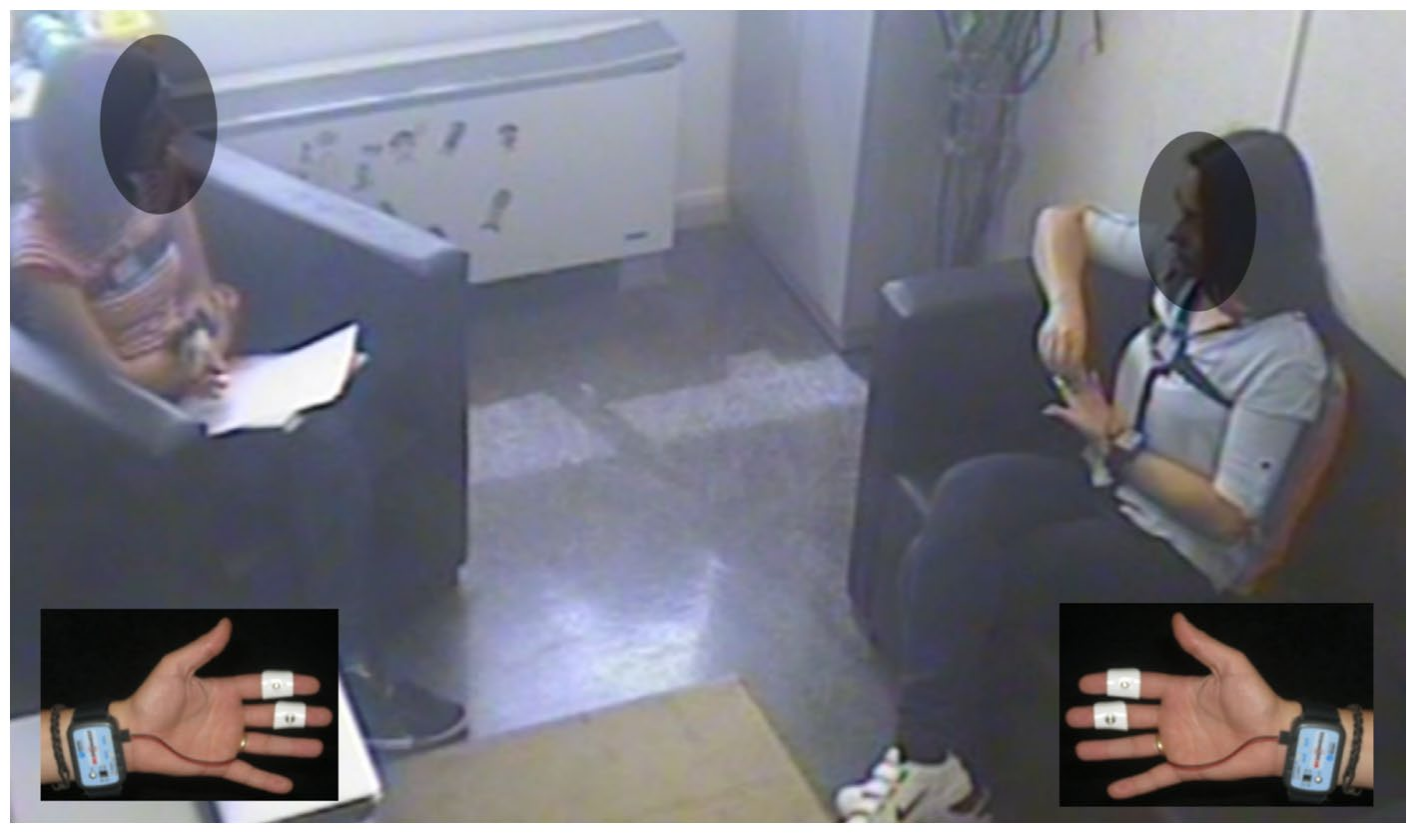
Still photo of a therapy session. Inserts: electrodermal sensors.

The EDA measurement was exosomatic based on the passage of an external direct current (DC) across the skin. Participants’ skin conductance (units in Microsiemens) was detected using a pair of pre-gelled disposable silver–silver chloride (Ag–AgCl) electrodes with a circular contact area of 1 cm diameter and isotonic gel (EL-507; BIOPAC Systems Inc., Santa Barbara CA, USA), which minimizes any interactions between skin and electrolyte. The electrodes were attached to the volar surfaces of the medial phalanges of the index and middle fingers of the nondominant hand. EDA was acquired using the modular system BIOPAC MP-150, which was connected to two electrodermal amplifiers, enabling the registration of both the patients’ and therapists’ electrodermal activity throughout the sessions. The system was connected to a computer, which enabled real-time registration of both participants using the AcqKnowledge 4.3 software of BIOPAC (Figure 1).

The present study employed an artifact detection algorithm for processing EDA signals, combining temporal consistency checks with adaptive thresholding. The methodology utilizes a sliding window approach (20 points at 2,000 Hz sampling rate, equivalent to 10 ms) to evaluate signal changes, which was chosen to capture sudden artifacts while preserving relevant physiological responses. The detection threshold was dynamically determined using the 99th percentile of the signal differences, making the approach adaptive to individual recording characteristics and more robust than fixed thresholds. This accounts for the inherent variability in physiological responses while effectively identifying common artifacts such as movement-related disturbances, electrode disconnections, and electrical interference. The conservative nature of the algorithm ensures that only clear artifacts are removed while preserving meaningful physiological changes. Manual supervision was added to confirm that only artifactual signals were identified using the described approach. Artifact detection was conducted using Python-based data processing tools within the computational environment JupyterLab. The preprocessing pipeline comprised specialized libraries including pandas (for data manipulation), NeuroKit2 (for physiological signal processing), bioread (for handling Biopac.acq files) and Plotly (for data visualization).

Once the raw data of electrodermal activity was obtained and further preprocessed using a 1 Hz low-pass filter, the sampling rate was reduced using the AcqKnowledge 4.3 software and set to 20 Hz. The resulting time series of electrodermal activity represent its phasic component (also called skin-conductance response). Data were exported as text files for ensuing synchrony analyses.

### 2.3 Procedures

The data utilized in this study originate from a larger research project investigating the physiological correlates of therapeutic collaboration (BIAL Foundation). This project was approved by the Ethics Committee of the Research Center in Psychology at the University of Minho (# CA_CIPsi-072012). Those patients who verbally consented to participate in this study were required to sign a consent form. This form informed patients of their obligations and rights, including that their personal treatment would continue should they decide to withdraw from the study at any timepoint. The psychotherapy sessions were provided at no cost. Prior to each session, both the patient and the therapist were prepared for the collection of physiological data. This data was monitored in real-time and recorded on a computer in a room adjacent to the consultation room, where research assistants supervised the recording process during the session. To establish the baseline values of physiological activity, participants were required to complete a minimally demanding baseline task, which involved describing neutral images that were presented on a monitor. This task lasted for approximately 10 min.

After the baseline task, participants filled out the symptom questionnaire (i.e., the OQ-45 in the initial session and the OQ-10 in all later sessions). Subsequently, the therapeutic session commenced, with an average duration of 50 to 60 min. At the end of each session, the patient and therapist completed the Working Alliance Inventory (WAI) and the Session Evaluation Questionnaire (SEQ). All data were appropriately coded to ensure patient confidentiality. The treatment plan comprised 16 sessions in accordance with the Cognitive Behavior Therapy protocol adopted. In some cases, the treatment was terminated at an earlier stage whenever the therapist and patient considered that the treatment goals had already been reached.

#### 2.3.1 Computation of synchrony

The EDA time series were analyzed using the R package SUSY (Tschacher and Haken, 2019; Tschacher, 2022) to quantify the overall synchrony between therapist’s and patient’s electrodermal activity in each session. SUSY is based on cross-correlations of the time series of each session, where cross-correlations are computed with time-lags up to ±5 s. These correlations capture simultaneous (lag = 0) as well as moderately time-lagged synchronization of the EDA values. Thus, the amount of synchrony both of simultaneous activity as well as of activity with response delays of up to 5 s is contained in the aggregated cross-correlations between therapist and patient.

To arrive at a control condition, surrogate tests are integrated in the SUSY algorithm: the time series of each session are segmented into 30-s intervals, and cross-correlations are computed and aggregated across all lags in each segment, and then averaged over all segments of the whole session. In the surrogate tests, this same procedure is repeated but with randomly shuffled segments, so that cross-correlations are computed on therapist’s and patient’s time series data that did not actually take place at the same time, providing a value of “pseudo-synchrony.” Such shuffling is repeated many times, which creates a distribution of pseudo-synchronies against which the real synchrony of a session is contrasted. This contrast is expressed by an effect size (ES) measure of synchrony for each session: ES = (real synchrony − mean pseudo synchrony)/standard deviation of pseudo synchronies. In accordance with the literature, we computed two effect sizes for each session: ES_abs_ and ES_noabs_. ES_abs_ is provided when all cross-correlations are taken as absolute values, that is with the minus sign of negative cross-correlations removed. The use of absolute values was the default in most early research on synchrony in psychotherapy (e.g., Ramseyer and Tschacher, 2011; Paulick et al., 2018). ES_noabs_ uses no absolute values and thus allows for so-called anti-phase synchrony (predominantly negative cross-correlations), where more EDA activity in one person is systematically coupled with less EDA activity in the other, hence negative ES_noabs_.

Cross-correlations are a function of the lags between the patient’s and therapist’s EDA time series. This cross-correlation function need not have symmetrical shape: If one person is the initiator of (here electrodermal) activity during the interaction, whereas the other person is largely responsive, this can be detected in the shape of the function, e.g., by higher cross-correlations when lags are negative than when they are positive. Thus, the lags of the cross-correlations that together form the overall measure of synchrony allow for a differentiation between the synchrony component in which the patient assumes the leading role and the synchrony component in which the leading role is with the therapist. Consequently, we computed a value of “patient-leading synchrony” considering only the cross-correlations of lags < 0 (i.e., patient leading) and a value of “therapist-leading synchrony” considering only lags > 0.

#### 2.3.2 Statistical analyses

To assess hypothesis 1, we conducted one-sample *t*-tests of the effect sizes ES_abs_ and ES_noabs_, which represent the patient-therapist synchronies of each session, against the null hypothesis that the synchronies would be absent, that is zero. Since we are dealing with a hierarchical dataset (sessions nested in therapy courses with the same patient and therapist), we repeated the one-sample *t*-tests also on the mean values per therapy course. The differences between the patient-leading and the therapist-leading synchrony components were assessed by pairwise *t*-tests. In consideration of the hierarchical dataset, we additionally used cluster-wise centering of the respective patient-and therapist-leading variables to account for the potential dependency structures, and applied the pairwise *t*-tests also on these centered variables.

The second, exploratory, research question of predominant in-phase or anti-phase synchrony was addressed by the results of hypothesis 1 concerning ES_noabs_. Evidence of in-phase synchrony was indicated when the mean value of ES_noabs_ would be significantly larger than zero. The associations between synchrony, alliance and outcome measures were analyzed using hierarchical regression models (“mixed-effects models”), in which level 1 was given by the values of sessions and level-2 by the 21 different therapy courses, in which the sessions were nested. Therapy# was therefore entered as the random effect in these hierarchical models, the synchrony variables ES_abs_, ES_noabs_, patient-leading ES_abs_, and patient-leading ES_noabs_ were used as the dependent variables. The variables of session-wise outcome (SEQ and OQ-10) and alliance scales (WAI) were the predictors (fixed effects) in these models. Ordinary regression models of only level 2 were used to analyze the link between therapy outcome and synchrony. OQ-45 changes provided the representations of outcome at the level of therapy courses and the mean synchrony aggregated across all sessions of the respective therapy course were used as the dependent variables.

## 3 Results

### 3.1 Synchrony and patient-leading synchrony

The synchronies of each of 299 sessions, sampled within 21 different therapy courses, were expressed by effect sizes ES_abs_ and ES_noabs_. The pair-wise Pearson correlation between ES_abs_ and ES_noabs_ was *r* = 0.22. We found overall significant synchronies across all sessions (Table 1) using one-sample *t*-tests against the null hypothesis of no synchrony, thus supporting Hypothesis 1 for both ES_abs_ and ES_noabs_. The expectation that patients and therapists would be synchronized with respect to their sympathetic activation was thus supported. The means of ES_noabs_ synchrony were significantly positive, which indicated that in-phase synchronization of therapists and patients clearly prevailed. Some sessions (17.7% of all sessions) yielded negative ES_noabs_, suggesting that in those sessions patient and therapist displayed anti-phasic physiological activations. Hypothesis 1 was also supported when one-sample *t*-tests were repeated on the means of ES_abs_ and ES_noabs_ per therapy course, thus removing the variance within therapy courses.

**Table 1:** Electrodermal synchronies of patient-therapist dyads across therapy sessions.

Synchrony Measure	*n*	*M*	*SD*	Lower CI	Upper CI	*p*
ES_abs_	299	0.31	0.60	0.24	0.38	*p*_0_ < 0.0001
ES_noabs_	299	14.14	21.17	11.73	16.55	*p*_0_ < 0.0001
ES_abs_(Pt)	299	0.45	0.90	0.35	0.56	*p*_2_ < 0.001
ES_abs_(Th)	299	0.20	0.87	0.10	0.30	
ES_noabs_(Pt)	299	28.94	65.70	21.47	36.42	*p*_2_ < 0.01
ES_noabs_(Th)	299	17.50	41.47	12.77	22.21	
mean ES_abs_	21	0.31	0.21	0.21	0.40	*p*_0_ < 0.0001
mean ES_noabs_	21	14.66	10.87	9.71	19.61	*p*_0_ < 0.0001
ES_abs_(Pt) centered	299	0.14	0.87	0.04	0.24	*p*_2_ < 0.001
ES_abs_(Th) centered	299	−0.11	0.86	−0.21	−0.02	
ES_noabs_(Pt) centered	299	14.83	62.23	7.75	21.91	*p*_2_ < 0.01
ES_noabs_(Th) centered	299	3.38	40.19	−1.19	7.95	

*n* = number of observations. *M* = mean. *SD* = standard deviation. *CI* = confidence intervals. *p*_0_ = *p-*values of one-sided *t*-tests, null hypothesis is “no synchrony is present in sessions.” *p*_2_ = *p-*values of pairwise *t*-tests patient-leading versus therapist-leading. ES_abs_ = Synchrony effect size per session using absolute cross-correlation values; ES_noabs_ = Synchrony effect size per session not using absolute cross-correlation values; ES_abs_(Pt), ES_abs_(Th), ES_noabs_(Pt), ES_noabs_(Th) = Synchrony effect sizes for patient-and therapist-leading. Mean ES_abs_, mean ES_noabs_ = means per therapy course. Centered = variables centered within clusters (clusters = 21 therapy courses).

With regards to leading and following of either patient or therapist, we separately considered only those lags in SUSY which mean that the patient’s EDA activation occurred before the activation of the therapist, hence labelled patient-leading synchrony. Using pairwise *t*-tests, it was found that patient-leading synchrony was significantly more pronounced than therapist-leading synchrony. Thus, patients tended towards showing a leading role in the sympathetic interactions during therapy sessions, whereas therapists tended towards responding physiologically to their patients. To account for the potential influences of dependency structures of this hierarchical dataset, we repeated the pairwise *t*-tests on variables that were centered cluster-wise (the clusters being the therapy courses). These analyses are also listed in Table 1. Analyses with centered variables replicated the finding of significantly higher patient-leading than therapist-leading synchrony.

### 3.2 Relationship between synchrony and the therapeutic alliance

Using hierarchical regression models, we analyzed the potential links of synchrony with questionnaire scales assessing the therapeutic alliance with respect to the therapeutic bond, agreement on tasks, and on therapy goals (WAI: Working Alliance Inventory). Synchrony ES_abs_ and ES_noabs_ (the latter distinguishing between in-phase and anti-phase synchrony) was not predicted by the WAI scales, yet patient-leading synchrony of both ES_abs_ and ES_noabs_ was. Models were computed first with the full set of predictors (e.g. all WAI scales), and then repeated with significant predictors only. If the repeated models turned out insignificant, they were not entered into the respective table. As is shown in Table 2, the quality of the therapeutic bond as assessed by therapists was positively linked with patient-leading synchrony. Qualitative analysis pointed to a single therapy course (Therapy# 16_05) out of the 21 therapies that had a large effect size towards anti-phase synchrony (mean ES_noabs_ of all its 12 sessions: −3.14). Here, anti-phase synchrony was present, especially in final sessions. In all recorded sessions of this therapy course, patient-leading was smaller than therapist-leading. Post-hoc inspection of this exceptional therapy showed higher than average ratings of the therapeutic bond despite anti-phase synchrony, which was counter to the statistical trend of the complete sample that overall showed in-phase synchrony related to bond ratings.

**Table 2:** Electrodermal patient-leading synchrony and therapeutic alliance scales.

Predictors	ES_abs_ (patient-leading)		ES_noabs_ (patient-leading)		ES_noabs_ (patient-leading)	
	Estimate	*t*	Estimate	*t*	Estimate	*t*
Intercept	−1.84	−1.38	−142.6	−1.85	−157.5	−2.19*
Bond-Pt	−0.04	−0.93	0.22	0.09		
Goal-Pt	0.05	0.94	−0.39	−0.13		
Task-Pt	−0.05	−1.02	−2.33	−0.87		
Bond-Th	0.15	2.08*	8.47	2.08*	8.77	2.34*
Goal-Th	−0.12	−1.92	2.32	0.63		
Task-Th	0.11	1.18	0.25	0.07		
Random effect						
Therapy-ID (% variance)	12.2		13.3		13.1	
*N*	286		286		288	
r^2^ (% variance)	18.8		17.9		17.3	

Results for hierarchical regression of patient-leading synchrony predicted by scales of the WAI. -Pt = scale rated by patient; -Th = scale rated by therapist. Only models containing significant predictors shown. *N*, number of observations. r^2^, explained variance of whole model. *p < 0.05.

### 3.3 Relationship between synchrony and session outcome

Further hierarchical regression models were implemented to test the associations between synchrony and the Session Evaluation Questionnaire (SEQ), with scales assessing ‘depth’ and ‘smoothness’ of sessions, as well as the patient’s ‘positivity’ and ‘arousal’. The scales were rated by both the therapist and patient after each session. Table 3 provides the results, showing negative links of synchrony with therapist-rated arousal (for synchrony based on absolute cross-correlations, ES_abs_). ES_noabs_ was positively associated with patient-rated depth and therapist-rated positivity. Patient-leading synchrony was not significantly predicted by SEQ scales. All hierarchical models listed in Tables 2 and 3 are random-intercept models. We computed also random-intercept plus random-slope models, which were however less appropriate throughout as judged by Akaike’s information criterium (AIC) and were not considered further.

**Table 3:** Electrodermal synchrony and session evaluation scales (SEQ).

Predictors	Synchrony ES_abs_		Synchrony ES_noabs_		Synchrony ES_abs_		Synchrony ES_noabs_	
	Estimate	*t*	Estimate	*t*	Estimate	*t*	Estimate	*t*
Intercept	−0.01	−0.03	−10.65	−0.60	0.95	4.56****	−25.06	−2.10*
Depth-Pt	0.06	1.37	3.43	1.98*			3.06	1.97
Smoothness-Pt	−0.03	−0.76	0.43	0.28				
Positivity-Pt	0.06	1.24	−1.57	−0.84				
Arousal-Pt	0.01	0.14	−1.97	−0.98				
Depth-Th	0.02	0.36	0.92	0.53				
Smoothness-Th	0.05	1.14	−1.28	−0.83				
Positivity-Th	−0.01	−0.15	4.47	2.01*			3.81	2.54*
Arousal-Th	−0.14	−2.28*	−1.31	−0.58	−0.16	−3.14**		
Random effect								
Therapy-ID (% variance)	1.2		22.5		2.2		17.0	
*N*	275		275		291		285	
r^2^ (% variance)	9.0		27.9		7.4		24.3	

-Pt = scale rated by patient; -Th = scale rated by therapist. Results for hierarchical regression of synchrony predicted by scales of the SEQ. Only models containing significant predictors shown. *N*, number of observations. r^2^, explained variance of whole model. *p < 0.05; **p < 0.01; ****p < 0.0001.

Hierarchical regression models of the associations between synchrony and the outcome questionnaire OQ-10 provided no significant associations between the scales wellness or distress and ES_abs_ or ES_noabs_. A significant negative association however existed between distress and patient-leading ES_abs_ (Table 4). Thus, in sessions with higher patient-leading ES_abs_, distress was attenuated.

**Table 4:** Electrodermal patient-leading synchrony and session evaluation scale ‘distress’ (OQ-10, rated by patient).

ES_abs_ (patient-leading)		
Predictors	Estimate	*t*
Intercept	0.73	2.97**
Distress	−0.05	−2.22*
Random effect		
Therapy-ID (% variance)	8.3	
*N*	276	
r^2^ (% variance)	14.4	

Results for hierarchical regression of synchrony predicted by ‘distress’. *N*, number of observations. r^2^, explained variance of whole model. *p < 0.05; **p < 0.01.

### 3.4 Relationship between synchrony and therapy outcome

The outcome questionnaire OQ-45.2, with subscales ‘social role’, ‘distress’ and ‘interpersonal relations’, was available as an overall outcome measure at the beginning (pre) and termination (post) of 19 therapy courses. The pre-post changes of subscale values were used as an estimation of outcome. One-sample *t*-tests against the null hypothesis “no change” were highly significant, pointing towards patients’ overall improvement. We conducted regression analyses assessing the associations between synchrony averaged across all sessions of a therapy course (the dependent variable) and such pre-post changes (the predictors). None of the pre-post changes were found significantly predictive of the average synchrony variables of the therapies.

## 4 Discussion

The present study aimed at analyzing the physiological coordination of patients and therapists in cognitive-behavioral psychotherapy sessions by addressing the synchrony of patients’ and therapists’ phasic component of electrodermal activity (EDA), a purely sympathetic signal. We also studied the association between EDA synchrony and the quality of the therapeutic alliance throughout the course of 21 therapeutic dyads across a total of 299 sessions. We specifically intended to empirically demonstrate the existence of EDA synchrony in the therapeutic dyadic interaction, as well as analyze the polarity of this co-variation (whether the observed synchrony was in-phase or anti-phase) and determine whether one of the two individuals of the dyad (the therapist or the patient) was leading the synchronized co-variation. Finally, we wished to illuminate how synchrony impacted both the quality of the alliance and session outcome.

We found support for our first research hypothesis, which postulated the existence of EDA synchrony in the therapeutic dyad on a session-wise basis, confirming that patients and their therapists were synchronized with respect to their sympathetic activity. We also verified that this synchrony was mostly in-phasic, finding in-phase synchronization of therapists and patients clearly prevailing. We found indications of in-phase synchrony associated with an in-depth therapeutic process and with patients’ emotional positivity. High synchrony was additionally linked with lowered emotional arousal of patients.

As regards the symmetry of the influence in the co-regulation of electrodermal responses, we found that patient-leading synchrony was significantly more pronounced than therapist-leading synchrony. Patients tended to show a leading role in the sympathetic activation during therapy sessions, whereas therapists tended more towards acting physiologically responsive to their patients. Importantly, this patient-leading synchrony was positively linked with the therapeutic bond as assessed by therapists. This seems to reflect the process in which the patient increases or decreases his or her own arousal, and the therapist mirrors these changes in a process of sympathetic-emotional contagion. As suggested by previous work, these affective dimensions of empathic processes are key in the affective or “bond” component of the alliance (Koole and Tschacher, 2016; Kykyri et al., 2019; Tourunen et al., 2022; Wiltshire et al., 2020). Our findings shed new light on the physiological processes underlying the therapeutic bond: we found the bond connected to in-phasic sympathetic synchrony where the patient assumes a leading role and the therapist follows after, hence reflecting the patient’s physiology in the therapist’s own body. This suggests embodied mechanisms of physiological interpersonal coordination that represent the fundamental empathic dimension of the therapeutic relationship. The findings are consistent with an “Archimedean role” of the therapist as an “unmoving mover” (Tschacher and Haken, 2019), a theoretical conception of the alliance which emphasizes the slower dynamics on the side of the therapist. This concept derives from the transdisciplinary theory of complexity science (‘synergetics’: Haken, 2006) that the slowly responding components of a system entrain the fast components, thereby leading to pattern formation. Therefore, the dynamics of the slow components start to govern the dynamics of all other components of the system. Applied to psychotherapeutic contexts, if the therapist constitutes the slow component, here in terms of sympathetic variability, he or she eventually shapes the dynamics of the complete therapy system, the therapist-patient alliance. Therapist-following and allowing for patient-leading may therefore be an important “therapist effect” (Lutz and Barkham, 2015) that augments therapists’ effectiveness.

These suggested mechanisms are also consistent with our finding of a negative association between patient’s distress and patient-leading synchrony. In other words, in sessions with higher patient-leading synchrony the patient’s distress was attenuated. Again, this emphasizes the importance of the therapist’s ability to be responsive to the patient by following in an interpersonal dance in which autonomic arousal is continually changing. This may be viewed as a physiological grounding of Rogerian client-centered theories regarding helping relationships, which are based on congruence and empathic understanding.

Limitations of the present analyses are connected to the hierarchical nature of the data set. Whereas the high sample size of sessions analyzed was satisfactory, the number of therapy courses with documented therapy outcome was insufficient and lacked statistical power to reliably test the association of synchrony with overall therapy outcome. This was corroborated by post-hoc power analyses that indicated appropriate sample sizes at the session level, but insufficient power for the test of overall therapy outcome.

### 4.1 Future directions

The advancement of the research on synchrony in psychotherapy would benefit from a consensus in the field on methodological procedures, which may be based either on cross-lagged correlations as in this article, or on regressions, or on recurrence techniques, or on frequency decomposition. Computing synchrony with substantially diverging algorithms can lead to widely varying, even contrary, results. Even the difference between absolute and non-absolute cross-correlations within the same approach is considerable, as the present analyses have shown; their correlation in the current dataset means they had only about 5% of shared variance. Therefore, several authors have argued for the definition of and adherence to shared and replicable empirical procedures (Altmann et al., 2022; Bar-Kalifa et al., 2019; Kleinbub, 2017; Palumbo et al., 2017), such as the present implementation of the surrogate-synchrony algorithm that is freely available as an R package.

Another important line of future research is the study of multimodality. First, the interpersonal synchrony in different therapeutic modalities should be compared to better understand whether the specificities of each theoretical orientation of psychotherapy may impact the association between synchrony and alliance or outcome. Second, a wide range of variables have been used to explore synchrony in psychotherapy: respiratory, cardiac and electrodermal peripheral physiological signals, body movement and eye movement, prosodic variables, and even central-nervous measures (the latter however still largely depend on laboratory settings and are thus less appropriate for psychotherapy research). As yet it is not known how the different modalities of synchrony relate to each other.

Finally, to complement the session-wise approach that we used in this study, future work on interpersonal synchrony should also apply microanalytic approaches focused on specific meaningful events of durations shorter than the whole duration of a therapy session. There is considerable variation of alliance quality on a smaller time scale than the 50 min of a therapy session, hence alliance ruptures and repairs should be considered and their potentially characteristic synchrony signatures elaborated (Høgenhaug et al., 2024). It may be of interest to study whether alliance ruptures are accompanied by anti-phasic synchrony with short duration. This implies methodological adaptations of the present algorithms towards also representing phasic synchrony alterations. Such a change of time scale is generally supported by nonverbal signals of therapeutic interaction and has become available by applying motion capture and the use of wearable physiological sensors, which provide data with high temporal resolution.

## Data Availability

The data supporting the conclusions of this article will be made available by the authors under the terms of the ethics approval, without undue reservation.
